# Nanostructure in Amphiphile-Based Deep Eutectic Solvents

**DOI:** 10.1021/acs.langmuir.3c02105

**Published:** 2023-11-15

**Authors:** Iva Manasi, Ralf Schweins, Kun Ma, Karen J. Edler

**Affiliations:** † Department of Chemistry, University of Bath, Claverton Down, Bath BA2 7AX, U.K.; ‡ Institut Laue-Langevin, CS 20156, Grenoble Cedex 9 38042, France; § ISIS Neutron and Muon Source, STFC, Rutherford Appleton Laboratory, Didcot OX11 0QX, U.K.; ∥ Department of Chemistry, Centre for Analysis and Synthesis (CAS), Lund University, Lund 221 00, Sweden

## Abstract

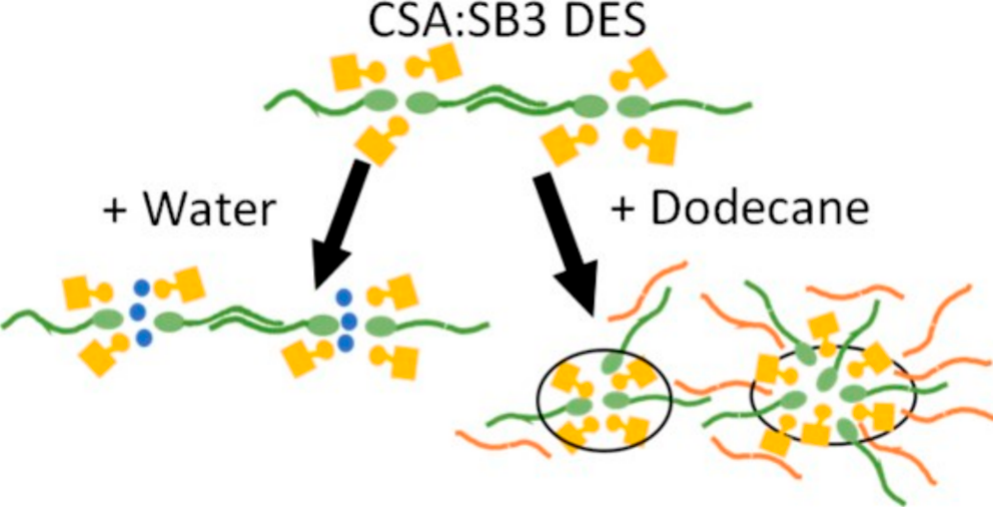

Deep eutectic solvents (DESs) are an emerging class of modern, often “green” solvents with unique properties. Recently, a deep eutectic system based on amphiphilic surfactant *N*-alkyl-*N*,*N*-dimethyl-3-ammonio-1-propanesulfonate (C12 & C14 sulfobetaine) and (1*S*)-(+)-10-camphor-sulfonic acid in the molar ratio 1:1.5 has been reported. Nanostructuring can be expected in this DES due to the nature of the components. In this work, we have investigated the native nanostructure in the DES comprising C12–C18 alkyl chain sulfobetaines with camphor sulfonic acid and how it interacts with polar and nonpolar species, water and dodecane, respectively, using small angle neutron scattering. By using contrast variation to highlight the relative position of the solvent components and additives, we can resolve the structure of the solvent and how it changes upon interaction with water and dodecane. Scattering from the neat DES shows structures corresponding to the self-assembly of sulfobetaines; the size of the structure increases as the alkyl chain length of the sulfobetaines increases. Water and dodecane interact, respectively, with the hydrophilic and hydrophobic moieties in the DES structure, primarily the sulfobetaine, thereby swelling and solvating the entire structure. The extent of the shift of the peak position, and the swelling, depend on concentration of the additive. The solution phase organization and the interaction of polar and nonpolar species as observed here, have the potential to affect the ordering of inorganic or polymeric materials grown in such solvents, paving new avenues for templating applications.

## Introduction

Deep eutectic solvents (DESs) are extensively hydrogen-bonded molecular solvents, eutectic mixtures of hydrogen bond donors (HBDs) and acceptors (HBAs) with low melting points.^1,2^ They share many features with ionic liquids (i.e., tunable physicochemical properties) which makes them viable green solvents that are less toxic than typical ionic liquids (ILs). Much like ILs, DESs are green solvents with low vapor pressure and a tunable nature; the hydrophobicity and physicochemical properties of the solvent can be altered by changing the DES components or with various additive compounds.^3,4^ Due to their tunability, these solvents continue to gain significant interest in various applications including, but not limited to, synthesis of organometallic compounds, porous materials and polymers,^5−7^ electroplating,^8^ drug, and pesticide delivery.^9−11^

**Figure 1:**
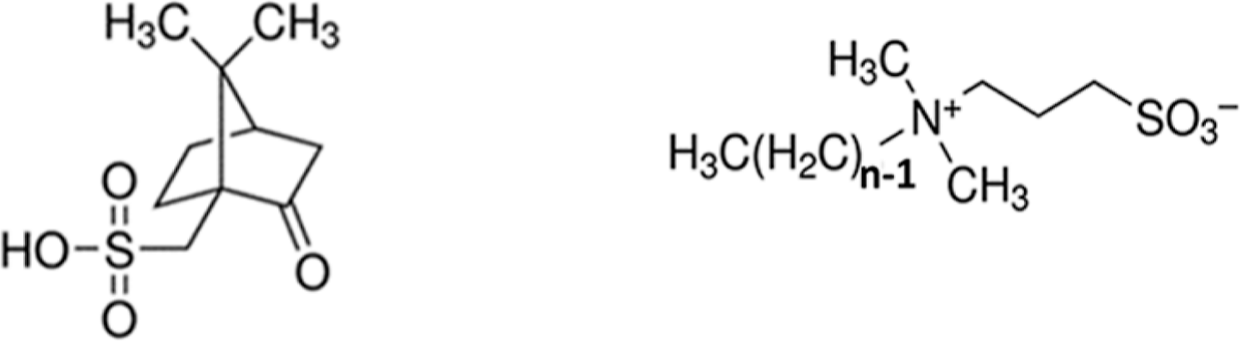
Chemical composition of the DES components: (1*S*)-(+)-10-camphor-sulfonic acid (left) and *N*-alkyl-*N*,*N*-dimethyl-3-ammonio-1-propanesulfonate with *n* = 12, 14, 16, or 18 (right).

Recently, a deep eutectic system based on amphiphilic surfactant *N*-alkyl-*N*,*N*-dimethyl-3-ammonio-1-propanesulfonate (C12 & C14) and (1*S*)-(+)-10-camphor-sulfonic acid in the molar ratio 1:1.5 has been reported by Cardellini et al.^12^ They investigated differently structured sulfobetaines, from aliphatic and aromatic to branched, and found that they all form room temperature eutectic mixtures at a molar ratio of 1 sulfobetaine to 1.5 or 2 acid. These are highly viscous solvents, with viscosity ∼3 Pa s at temperatures as high as 85 °C, with melting points below 0 °C. Cardellini et al. investigated the physicochemical properties of the DES, the toxicity of these mixtures on a eukaryotic model, and the potential use of these DES as Brønsted catalyst media for synthesis application. However, they did not investigate the structure or the potential application of these DES as a medium for nanostructure-driven templating applications. Nanostructuring can be expected in these solvents due to the nature of their components (see Figure 1). The interaction of randomly oriented nanostructured domains may indeed be the cause of their extremely high viscosity. However, these DES offer unique potential in terms of exploiting solvent nanostructure for templating applications. Similar structures have been found in ionic liquids where salt components contain long alkyl chains,^13^ but so far, little work has been done to exploit such solvent nanostructures in materials synthesis or to understand how the structuring might affect the solubility of other species in such solvents. Such solution phase organization may affect the ordering of inorganic or polymeric materials grown in such solvents, by templating or via solute orientation at the internal interfaces which could affect reactivity. In this preliminary work, we have explored the native nanostructure in the DES and how it interacts with exemplar polar and nonpolar species, water and dodecane, respectively. We have studied an additive-in-DES concentration range relevant to templating applications, although we note that the DES is soluble in both species so adding sufficient of either water or dodecane will form DES-in-solvent structures that are not of interest here. To probe the formation of self-assembled structures in these DES, we take advantage of neutron contrast to highlight the relative position of solvent components vs additives.

## Methods and Materials

### Materials

(1*S*)-(+)-10-Camphor sulfonic acid (C_10_H_16_O_4_S; CSA; 99%), *N*-dodecyl-*N*,*N*-dimethyl-3-ammonio-1-propanesulfonate [CH_3_(CH_2_)_11_N^+^(CH_3_)_2_CH_2_CH_2_CH_2_SO_3_^–^; SB3-12; ≥99%], *N*-tetradecyl-*N*,*N*-dimethyl-3-ammonio-1-propanesulfonate [CH_3_(CH_2_)_13_N^+^(CH_3_)_2_CH_2_CH_2_CH_2_SO_3_^–^; SB3-14; ≥99%], *N*-hexadecyl-*N*,*N*-dimethyl-3-ammonio-1-propanesulfonate [CH_3_(CH_2_)_15_N^+^(CH_3_)_2_CH_2_CH_2_CH_2_SO_3_^–^; SB3-16; ≥98%], dodecane (C_12_H_26_; h-Dodec; ≥99%), and deuterium oxide (D_2_O; 99.9 atom % D) were obtained from Sigma-Aldrich, UK. *N*-Octadecyl-*N*,*N*-dimethyl-3-ammonio-1-propanesulfonate [CH_3_(CH_2_)_17_N^+^(CH_3_)_2_CH_2_CH_2_CH_2_SO_3_^–^; SB3-18; ≥98%] was obtained from Fluka. Tail deuterated SB3-12 [CD_3_(CD_2_)_11_N^+^(CH_3_)_2_CH_2_CH_2_CH_2_SO_3_^–^; d25-SB3-12, 98% D] and SB3-14 [CD_3_(CD_2_)_13_N^+^(CH_3_)_2_CH_2_CH_2_CH_2_SO_3_^–^; d29-SB3-14, 98% D] were supplied by the STFC ISIS Deuteration Facility. Perdeuterated dodecane (C_12_D_26_; d-Dodec; 98% D) was obtained from CK Isotopes Ltd.

### Sample Preparation

The CSA:SB3-Cn (Cn = 12, 14, 16, 18; HH DES) DES were prepared by combining the components in molar ratios of 1.5:1. These mixtures were stirred at 90 °C until a clear, homogeneous liquid was obtained, which was subsequently sealed. Only SB3-12 and SB3-14 were available in the tail deuterated form. For these two, partially deuterated DES (CSA:d25-SB3-12 and CSA:d25-SB3-14; HD DES) were prepared by combining the acid with the deuterated sulfobetaines in the molar ratio 1.5:1 and following the same procedure. DES mixtures with additives were prepared by mixing the required concentration (5, 10, and 20 wt %) of water, D_2_O, dodecane, or d-dodecane with the DES at 60 °C until a homogeneous solution was obtained. Preparation of the DES prior to the addition of water or dodecane allows small amounts of water to be easily added to the equilibrated liquid mixture, forming homogeneous solutions much more rapidly than addition to solid components.

### Methods

Differential scanning calorimetry (DSC) measurements to determine the melting/transition temperature of the neat DES were carried out on a TA Instruments DSC-Q20 differential scanning calorimeter. The sample was first equilibrated at −60 °C and held for 5 min, heated to 60 °C at a ramp rate of 5 °C min^–1^, and held for 5 min, before cooling to −60 °C at the same ramp rate. The transition temperature was measured from the change in the baseline.

Flow curves for the DES and DES with added water or dodecane were measured using a TA Instruments HR-3 Discovery hybrid rheometer operating in a cone (1.00944°) plate geometry with a temperature of 70 °C using a Peltier control. The stress response of the samples was measured for the applied shear rate ranging from 0.1 to 100 s^–1^. Viscosity of the samples was calculated from the shear stress versus rate response.

Small angle X-ray scattering (SAXS) was measured using an Anton Paar SAXSpoint 2.0 with Cu Kα radiation (λ = 1.5418 Å) giving a *q*-range of 0.01 Å^–1^ < *q* < 0.42 Å^–1^. Wide angle X-ray scattering (WAXS) measurements were also done on the SAXSpoint 2.0 at the same time by changing the sample–detector distance, giving a *q*-range of 0.1 Å^–1^ < *q* < 2.0 Å^–1^. Small angle neutron scattering (SANS) measurements were carried out on the D22 SANS instrument at the Institut Laue-Langevin, France (experiment no. 9-10-1699^14^) with a *q*-range of 0.0045–0.66 Å^–1^. The samples were loaded into 1 mm path length rectangular quartz cuvettes (Hellma GmbH, Germany) and placed on the automatic sample changer on the instrument and measured at 70 °C. Data reduction was performed according to the standard procedures, resulting in output converted to absolute units of the scattering intensity [*I*(*q*), cm^–1^] vs the momentum transfer (*q*, Å^–1^). Subtraction of the scattering from the empty cuvettes was performed and the data was analyzed using the NIST NCNR macros in Igor Pro^15^ and SASVIEW.^16^ A description of the models used for fitting the data is given in the Supporting Information.

## Results and Discussion

### DES Characterization

A summary of the glass transition temperature and viscosity measurements carried out on the DES is given in Table 1. DSC measurements carried out on the CSA:SB3-Cn DES are detailed in the Supporting Information and shown in Figure S1. Using these measurements, a glass transition temperature (*T*_g_) of −19.4 °C can be estimated for CSA:SB3-12, −20.8 °C for CSA:SB3-14, −22.5 °C for CSA:SB3-16, and −18.5 °C for CSA:SB3-18. The glass transition temperature decreases as the alkyl chain length of the surfactant increases from C12 to C16. This is consistent with the results on melting point observed by Cardellini et al. for CSA:sulfobetaine DES with varying alkyl chain length.^12^ However, we see a rise in *T*_g_ for SB3-18, which could possibly suggest that the mixture is off its eutectic point. A change in eutectic molar composition has been observed in case of menthol and carboxylic acid-based hydrophobic DES where more menthol is required as the alkyl chain length of the carboxylic acid increases to reach the eutectic point.^17^ However, in this particular study, we have kept the molar ratio of CSA:SB3-Cn as 1.5:1 for all sulfobetaine alkyl chains, to remain consistent in our comparison between different DES.

**Table 1:** Glass Transition Temperature (*T*_g_), Viscosity (η) without and with Added 10 wt % Water and Dodecane for the Different CSA:SB3-Cn DES

DES	*T*_g_ (°C)	η (pure DES) (Pa s)	η (+10 wt % H_2_O)^a^ (Pa s)	η (+10 wt % Dodec)^a^ (Pa s)
CSA:SB3-12	–19.4	4.6 ± 0.11	0.8	2.3
CSA:SB3-14	–20.8	4.5 ± 0.10		
CSA:SB3-16	–22.5	5.5 ± 0.12		
CSA:SB3-18	–18.5	6.0 ± 0.19	1.2	3.6

a The viscosity vs shear rate graph for CSA:SB3-Cn DES with water and dodecane show non-Newtonian behavior, especially below shear rates of ∼1 s^–1^. Therefore, the viscosity specified here is at a shear rate of 10 s^–1^, at which point a Newtonian plateau is reached.

Viscosity measurement carried out on the CSA:SB3-Cn DES for different alkyl chain lengths of the sulfobetaine are detailed in the Supporting Information and shown in Figure S2. There is no shear rate dependence of the viscosity for any of the four DES studied, and the average viscosity for the DES at 70 °C are 4.6 ± 0.11 Pa s for CSA:SB3-12, 4.5 ± 0.10 Pa s for CSA:SB3-14, 5.5 ± 0.12 Pa s for CSA:SB3-16, and 6.0 ± 0.19 Pa s for CSA:SB3-18. The viscosity increases as the alkyl chain length of the sulfobetaine in the DES increases, an effect also seen in ionic liquids for alkyl chains with *n* > 4.^18−22^ There the viscosity increase was attributed to the presence of heavier and bulkier ions in the ionic liquids containing longer aliphatic chains. A similar effect is also observed for hydrophobic eutectic solvents based on terpenes and monocarboxylic acids.^17^ Contrary to our measurements, Cardellini et al. report a decrease in viscosity (at 85 °C) of CSA:SB3-Cn DES upon increasing the alkyl chain length of the sulfobetaine; 5.371 Pa s for CSA:SB3-4, 3.025 Pa s for CSA:SB3-12, and 2.161 Pa s for CSA:SB3-14.^12^ They do not compare these three SB3 sulfobetaines to each other in their discussion and instead primarily use the SB3-Cn data to discuss comparisons between sulfobetaines having similar tail lengths but differing groups on the ammonium (methyl, ethyl, and propyl) as well as different linker lengths in the headgroup changing from C3 to C4. Differences in purity and moisture absorption may be a reason for this difference in viscosity between our and their measurements. In addition to affecting the viscosity of the mixtures, the alkyl chain is expected to result in nanostructuring within the liquid, and this is further investigated using SANS and SAXS.

**Figure 2:**
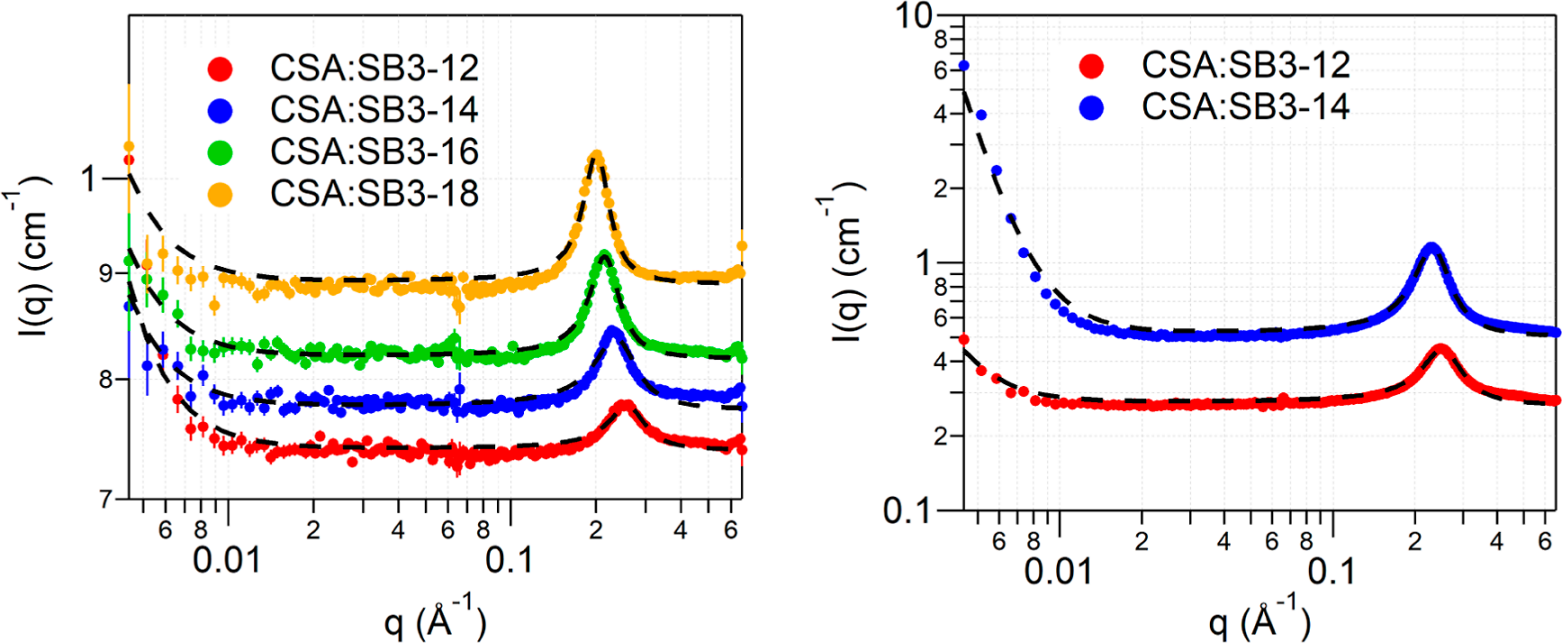
SANS data from CSA-SB3-Cn DES at HH contrast (left) and HD contrast (right) with the CSA:SB3-12 shown in red, CSA:SB3-14 in blue, CSA:SB3-16 in green, and CSA:SB3-18 in yellow. The data is fitted to a broad peak model (black dashed lines). The SANS patterns in the figures are offset along the *y*-axis for clarity.

SANS data were collected on h-CSA:h-SB3-Cn (HH DES; SB3-12, SB3-14, SB3-16 and SB3-18) and h-CSA:d-SB3-Cn (HD DES; d25-SB3-12 and d29-SB3-14) DES and are shown in Figure 2. SAXS data was also collected on all of the above samples and are shown in Figure S4. The data shows a characteristic peak at *q* ∼ 0.2 Å^–1^ as well as Porod ∼ *q*^–4^ scattering, ascribed to micro air bubbles in these highly viscous DES, at *q* < 0.01 Å^–1^. The characteristic peak shifts to lower *q* as the alkyl chain length of sulfobetaine increases. WAXS data collected at the same time shows no higher order peaks related to the first peak (Figure S6 left panel). A broad scattering feature exists between 1.2 and 1.5 Å^–1^, which is a typical “adjacency” peak arising from overall intermolecular and intramolecular interactions between adjacent atoms that exist in all fluids regardless of their polarity.^23,24^ The HH and HD neutron contrast and the SAXS measurement give the same peak positions within measurement resolution. The data was fitted to a broad peak model and the peak positions are summarized in Table 2. Details of data fitting can be found in the Supporting Information. The peak positions, averaged from the SANS and SAXS measurements, correspond to *d*-spacing of 24.8 Å for SB3-12, 27.1 Å for SB3-14, 29.2 Å for SB3-16, and 31.3 Å for SB3-18. The *d*-spacing shows a linear dependence on the alkyl chain length of the sulfobetaine with an average increase of 1.1 Å per carbon atom in the alkyl chain. The *d*-spacing is smaller than would be expected for a sulfobetaine bilayer comprising the corresponding fully extended C12, C14, C16, and C18 alkyl chains,^25^ indicating intercalation of the tails. Using the sulfobetaine molecule length and the *d*-spacing from the scattering data, there is an average of 45% alkyl chain overlap in the bilayers. This large extent of interdigitation may contribute to the high viscosities measured for these liquids.

**Table 2:** SAXS and SANS Peak Position and Calculated *d*-Spacing for the CSA-SB3-Cn DES Samples

DES	peak position^a^				*d*-spacing = 2π/*q*_0_
	SANS HH contrast (Å^–1^)	SANS HD contrast (Å^–1^)	SAXS (Å^–1^)	average (Å^–1^)	(Å)
CSA:SB3-12	0.258	0.252	0.250	0.253	24.8
CSA:SB3-14	0.235	0.232	0.230	0.232	27.1
CSA:SB3-16	0.215		0.215	0.215	29.2
CSA:SB3-18	0.201		0.200	0.201	31.3

a The error in the determination of the peak position is <1%.

**Figure 3:**
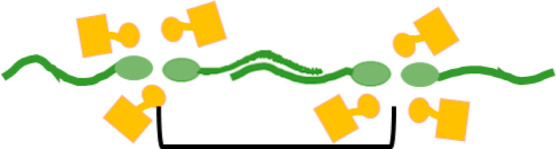
A schematic depicting the proposed DES structure with the CSA shown as yellow blocks and the sulfobetaine shown in green. The black brackets depict the repeating unit that gives rise to the *d*-spacing.

These measurements show clear nanostructuring in the DES, that can be attributed to the self-assembly of amphiphilic sulfobetaine to optimize interaction with the camphor sulfonic acid. Three factors could lead to nanosegregation in the CSA:SB3-Cn DES. Electrostatic attractions between the zwitterionic headgroup of the sulfobetaine and hydrogen bonding interactions between the headgroup and the sulfonic acid (of the CSA) will favor the creation of ionic/polar domains. Creation of polar domains will exclude the nonpolar parts of the molecules, promoting clustering of the alkyl groups into nonpolar domains. This kind of nanostructural organization is also observed in both short^26−33^ and long-chain ionic liquids^34−38^ and surfactant-based surface active ionic liquids (SAILS),^39−41^ where interdigitation of ionic and alkyl chain leads to distinct domains resulting in a sponge-like structure. The size of the nanostructure domains has a distinct dependence on the alkyl chain length of the sulfobetaine used to make the DES, suggesting a repeating domain comprising the sulfobetaine alkyl chains. The volume ratio of ionic/polar to the alkyl components is near unity (volume of 1.5 × sulfonic acid + sulfobetaine headgroup = 270 Å^3^; volume of C12 chains = 377 Å^3^), giving a packing parameter ∼1 and, therefore, a locally lamellar structure is most probable. A schematic of the CSA and sulfobetaine interaction and organization is depicted in Figure 3. Informed by similar amphiphilicity determined nanostructures in ionic liquids,^31^ the absence of higher order peaks in WAXS, and the lack of birefringence in the CSA:SB3-12 DES, we propose that the lamellar layering is quite disordered and the overall structure comprises a bicontinuous sponge-like structure.

### Addition of Water

**Figure 4:**
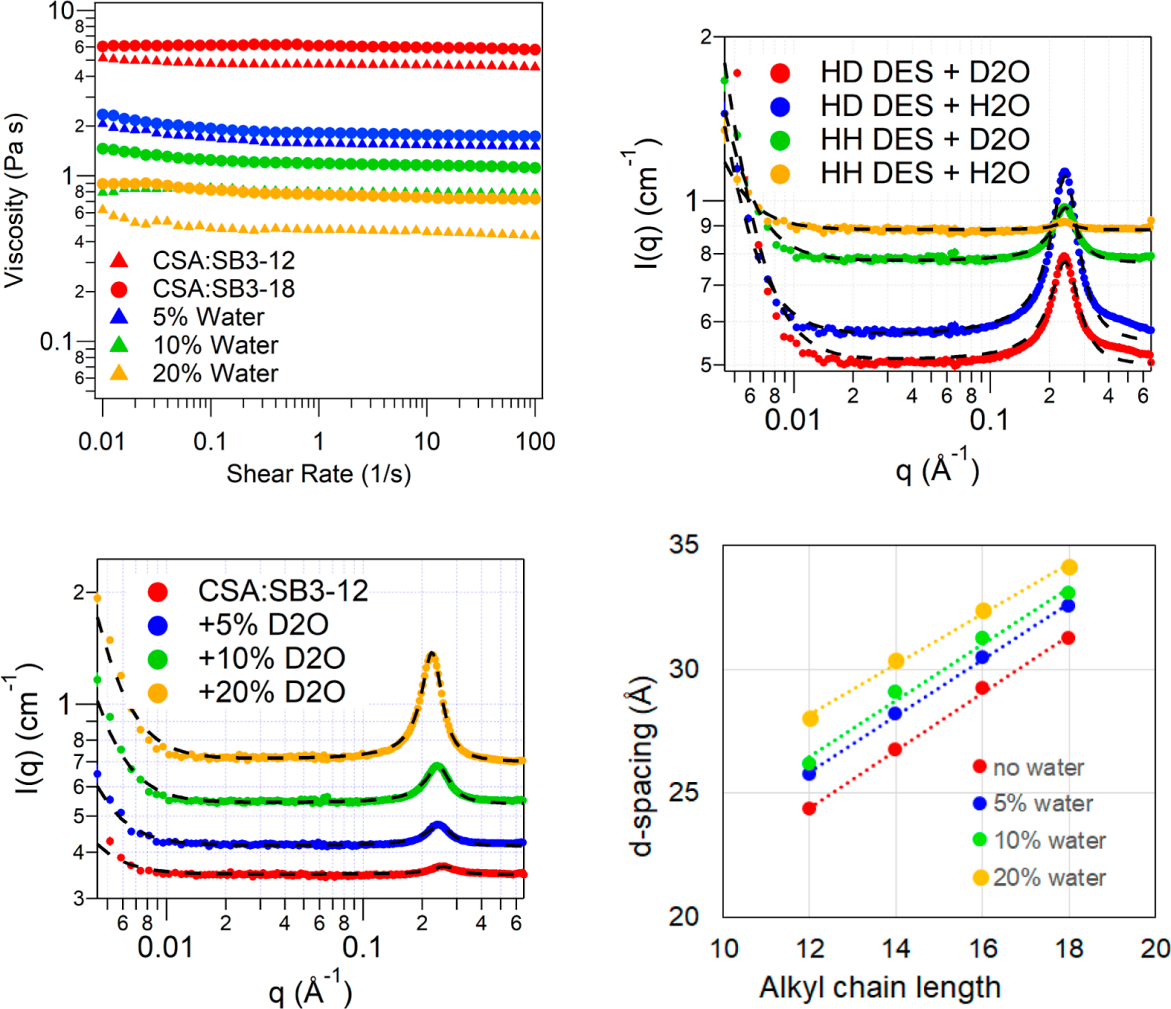
Top left: Shear rate vs viscosity data for the CSA:SB3-12 (denoted by triangles) and CSA:SB3-18 (denoted by circles) DES with added water (D_2_O) at three concentrations: 5 wt % (blue data set), 10 wt % (green data set), and 20 wt % (yellow data set) along with the neat DES (red data set). Top right: SANS data from 10 wt % added water in CSA:SB3-12 at 4 different contrasts: HD DES with D_2_O (red), HD DES with H_2_O (blue), HH DES with D_2_O (green), and HH DES with H_2_O (yellow). The data is fitted to a broad peak model (black dashed lines). Bottom left: SANS data from CSA:SB3-12 DES with added D_2_O at three concentrations: 5 wt % (blue data set), 10 wt % (green data set), and 20 wt % (yellow data set) along with the neat DES (red data set). The data is fitted to a broad peak model (black dashed lines). Bottom right: Calculated *d*-spacing for the different added water concentrations plotted vs the alkyl chain length of the sulfobetaines in the CSA:SB3-Cn DES. The error bars are smaller than the symbols since the uncertainties in the calculated *d*-spacings are <1% as for the Table 2. The SANS patterns in the top right and bottom left graphs are offset along the *y*-axis for clarity.

The effect of the addition of water on the viscosity of the CSA:SB3-Cn DES is shown in Figure 4 (top left panel). A strong dependence of viscosity on water concentration is characteristically observed for many DES and is used to overcome practical challenges posed by high DES viscosity in various applications.^4,42−47^ However, the addition of water to surfactant nanostructures can increase or decrease viscosity, depending on the structure,^48^ so we wished to quantify the effect of water addition to these amphiphilic DES. We measured the viscosity vs shear rate for the DES with addition of three concentrations of water (5, 10, and 20 wt %) for the two extreme chain lengths of the sulfobetaine in the DES (SB3-12 and SB3-18) and infer trends for the intermediate chain lengths based on these. The viscosity data at three shear rates (0.01, 10, and 100 s^–1^) is also summarized in Table S1. First, the addition of water reduces the viscosity drastically; the high-shear viscosity (shear rate = 10 s^–1^) of CSA:SB3-12 was reduced from 4.6 Pa s for neat DES to 1.5 Pa s for 5 wt % added water to 0.46 Pa s for 20 wt % added water and of CSA:SB3-18 from 6.0 Pa s for neat DES to 1.8 Pa s for 5 wt % added water to 0.74 Pa s for 20 wt % added water. For the three measured data points, the viscosity shows a power law dependence on the water concentration for both CSA:SB3-12 and CSA:SB3-18 DES (Supporting Information Figure S3). Second, at low shear rates (<0.1 s^–1^), we observe that the viscosity decreases with increasing shear rate. This is typical non-Newtonian shear-thinning behavior and reflects changing of the interconnected sponge-like structure of the DES by the shearing action. However, in this case, the shear-dependence is not very strong; the viscosity reduces by ∼20% of its value at shear rate of 0.01 to 10 s^–1^, and, therefore, structural changes would also be small.

SANS was measured from h-CSA:h-SB3-Cn with D_2_O at the three different concentrations: 5, 10, and 20 wt %. The SANS data for D_2_O in CSA:SB3-12 is shown in Figure 4 (bottom left). The data for CSA:SB3-14, CSA:SB3-16, and CSA:SB3-18 DES with D_2_O is shown in Supporting Information Figure S7. As with the SANS patterns from the neat DES, the data from DES with D_2_O shows Porod ∼ *q*^–4^ scattering due to air microbubbles at *q* < 0.01 Å^–1^ and a characteristic peak at *q* ∼ 0.2 Å^–1^. The peak shifts to lower *q*, upon increasing the concentration of added D_2_O. The data was fitted to the broad peak model, and the calculated *d*-spacing, using the peak positions, is plotted in Figure 4 (bottom right). As can be seen from the graph for each sulfobetaine, the *d*-spacing increases with an increasing concentration of the D_2_O added. The extent of the shift of the peak position, and the swelling, depends on concentration of the water added, with the structure swelling by 3–4 Å upon the addition of 20 wt % D_2_O. For CSA:SB3-12, the *d*-spacing increases from 24.4 Å for neat DES to 25.8 Å for DES with 5 wt % D_2_O to 26.2 Å for DES with 10 wt % D_2_O to 28.0 Å for DES with 20 wt % D_2_O. All four DES studied here show a linear increase of the *d*-spacing with increasing water concentration.

To further investigate the changes in the nanostructure of the DES upon addition of water, four different contrasts of the DES/water mixture were measured for the C12 sulfobetaine: h-CSA:h-SB3-12 (HH DES) with 10 wt % added H_2_O & D_2_O and h-CSA:d-SB3-12 (HD DES) with 10 wt % added H_2_O & D_2_O. These are shown in Figure 4 (top right). SAXS was also measured from all of the above samples, Supporting Information Figure S5 (left). The SANS and SAXS data here show no further structural information. The SAXS data for the four contrasts are identical, and in the SANS patterns, there is a slight change in the overall and peak intensity based on the contrast in the mixture. The HH DES with added H_2_O shows the highest incoherent background and the lowest contrast with the HD DES with added D_2_O showing the lowest background and the HD DES with added H_2_O showing the highest intensity. The data are still best described by a broad peak model, and the peak positions for the different contrasts in both SAXS and SANS are same within the experimental resolution. WAXS data shows no additional structural features after water addition (e.g., Figure S6 right panel). The average *d*-spacing for 10 wt % added water in CSA:SB3-12 was found to be 26.2 Å from SANS and 26.6 Å from the SAXS.

**Figure 5:**
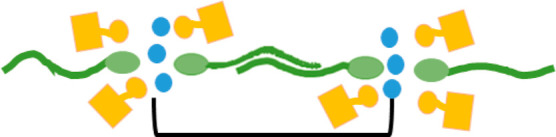
A schematic depicting the proposed DES structure with the CSA shown as yellow blocks, the sulfobetaine shown in green, and the water as blue circles. The black bracket depicts the repeating unit that gives rise to the *d*-spacing.

The SANS and SAXS data show that the addition of water to the DES increases the *d*-spacing between ordered sulfobetaine domains. The small water molecule incorporates in the DES nanostructure and swells the structure without disrupting it. Being a polar molecule, water can interact with and incorporate into the ionic/polar domains (sulfobetaine headgroup and CSA) as a solvation shell to the headgroups, thus increasing their volume and changing the packing parameter (packing parameter still remains ∼1) only slightly. Therefore, a locally lamellar structure still remains with an overall spongelike structure, and the solvation of the headgroup by the water molecule effectively pushes the entire domains apart or swells the structure, manifested as increase of the *d*-spacing calculated from the scattering curves. A schematic of this is depicted in Figure 5. A similar sponge-like structure is observed for the [BMIm][AOT] ionic liquid/water mixture for water concentrations of up to 20–40 vol % depending on the temperature. In that case also, the authors report swelling of the polar domains upon addition of small amounts of water, over a similar composition range to that used here. Further dilution of that system resulted in a lamellar arrangement (20–80 vol % water) and finally [BMIm][AOT] vesicles were observed for very dilute systems (80 vol % water).^49^

### Addition of Dodecane

**Figure 6:**
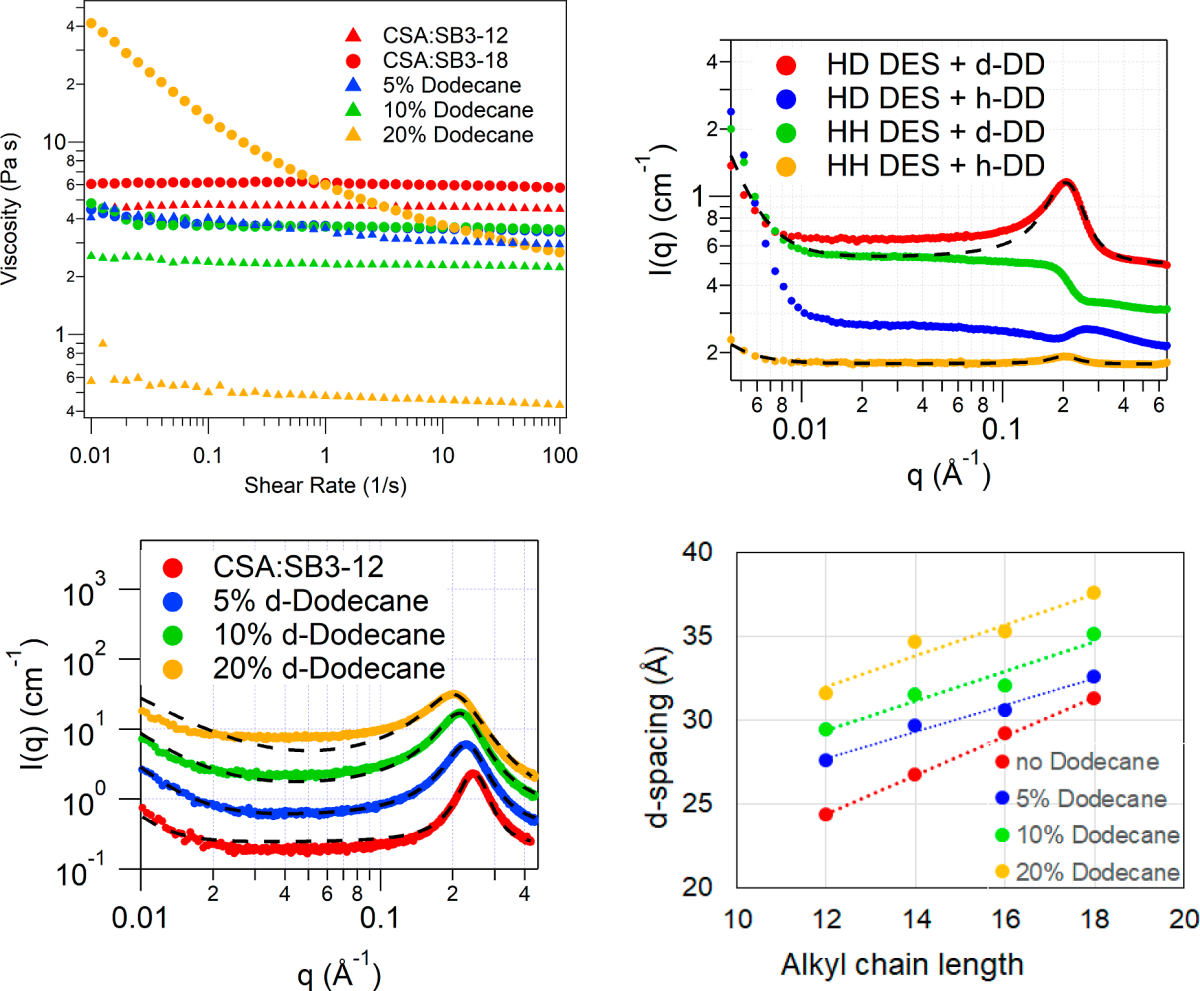
Top left: Shear rate vs viscosity data for the CSA:SB3-12 (denoted by triangles) and CSA:SB3-18 (denoted by circles) DES with added dodecane at three concentrations: 5 wt % (blue data set), 10 wt % (green data set), and 20 wt % (yellow data set) along with the neat DES (red data set). Top right: SANS data from 10 wt % added dodecane in CSA:SB3-12 at four different contrasts: HD DES with d-dodecane (red), HD DES with h-dodecane (blue), HH DES with d-dodecane (green), and HH DES with h-dodecane (yellow). The data is fitted to a broad peak model (black dashed lines). Bottom left: SAXS data from CSA:SB3-12 DES with added d-dodecane at three concentrations: 5 wt % (blue data set), 10 wt % (green data set), and 20 wt % (yellow data set) along with the neat DES (red data set). The data is fitted to a broad peak model (black dashed lines). Bottom right: Calculated *d*-spacing for the different added dodecane concentrations plotted vs the alkyl chain length of the sulfobetaines in the CSA:SB3-Cn DES. The error bars are smaller than the symbols since the uncertainties in the calculated *d*-spacings are <1% as for the Table 2. The SANS patterns in the top right and bottom left graphs are offset along the *y*-axis for clarity.

The effect of the addition of dodecane on the viscosity of the CSA:SB3-Cn DES is shown in Figure 6 (top left panel). Dodecane here is used as an exemplar nonpolar species to see how the DES structure and, therefore, properties are affected by interactions with nonpolar moieties. We measured the viscosity vs shear rate for the DES with addition of three concentrations of dodecane (5, 10, and 20 wt %) for the two extreme C12 and C18 chain lengths sulfobetaine in the DES. The viscosity data at three shear rates (0.01, 10, and 100 s^–1^) is also summarized in Table S1. Addition of dodecane also reduces the viscosity drastically though not quite to the same extent as water; the high-shear viscosity (shear rate = 10 s^–1^) of CSA:SB3-12 was reduced from 4.6 Pa s for neat DES to 3.1 Pa s for 5 wt % added dodecane to 0.46 Pa s for 20 wt % added dodecane and of CSA:SB3-18 from 6.0 Pa s for neat DES to 3.6 Pa s for 5 wt % added dodecane and thereafter remained constant (see Supporting Information Figure S3). Immediately two things are evident: first, upon addition of dodecane the high shear viscosity of SB3-12 shows a linear dependence with dodecane content, and second, for SB3-18 the high shear viscosity drops significantly upon addition of 5 wt % dodecane to overlap that of the SB3-12 system, and thereafter the change is slow and shear dependent. For CSA:SB3-12, the shear dependence of viscosity is small, like with water; a maximum of 20% reduction in viscosity is observed from low to high shear rates. On the other hand for dodecane in CSA:SB3-18, a strong shear-thinning non-Newtonian behavior is observed; for 5 wt % dodecane in CSA:SB3-18, the viscosity reduces by 20% of its value from a shear rate of 0.01 to 10 s^–1^; for 10 wt % dodecane in CSA:SB3-18, viscosity reduces by 25% from low to high shear rates; and for 20 wt % dodecane in CSA:SB3-18, the initial viscosity is much higher and the reduction in viscosity is of an order of magnitude from 41.4 Pa s at 0.01 s^–1^ to 3.7 Pa s at 10 s^–1^ and 2.7 Pa s at 100 s^–1^. These trends suggest that at low dodecane concentration, the changes in structure are small; however, at higher dodecane concentration large changes in structure can be expected, indicating a dependence of the structure on dodecane concentration.

SANS and SAXS were measured from h-CSA:h-SB3-Cn with d-dodecane at the three different concentrations: 5, 10, and 20 wt %. In this case, the SANS data show micellar scattering along with the broad peak. The SAXS data, where the scattering is dominated by the Porod scattering due to micro air bubbles at *q* < 0.01 Å^–1^, shows the characteristic broad peak at *q* ∼ 0.2 Å^–1^ and was used to get concentration trends, with further structural details investigated using the multicontrast SANS data. The SAXS data for d-dodecane in CSA:SB3-12 are shown in Figure 6 (bottom left). The SAXS data for CSA:SB3-14, CSA:SB3-16 and CSA:SB3-18 DES with d-dodecane are shown in Supporting Information Figure S8. The data was fitted to the broad peak model, and the calculated *d*-spacing, using the peak positions, is plotted in Figure 6 (bottom right). As can be seen from the graph for each sulfobetaine, the *d*-spacing increases with concentration of the dodecane added. The increase in the *d*-spacing and thereby the swelling is greater than that observed for water, consistent with the larger size of the dodecane molecule compared to water. The swelling of the structure depends on the concentration of the dodecane added, with the structure almost swelling by 6–8 Å upon the addition of 20 wt % dodecane. For CSA:SB3-12, the *d*-spacing increases from 24.4 Å for neat DES to 27.6 Å for DES with 5 wt % dodecane to 29.4 Å for DES with 10 wt % dodecane to 31.6 Å for DES with 20 wt % dodecane. All four DES studied here show a linear increase of the *d*-spacing with increasing dodecane concentration, although the rate of change is different for each DES (Figure 6, bottom right).

To further investigate the changes in the nanostructure of the DES upon addition of dodecane, four different contrasts of the DES/dodecane mixture were measured for the C12 sulfobetaine: h-CSA:h-SB3-12 (HH DES) with 10 wt % added h-dodecane and d-dodecane and h-CSA:d-SB3-12 (HD DES) with 10 wt % added h-dodecane and d-dodecane. These are shown in Figure 6 (top right). SAXS was also measured from all of the above samples, Supporting Information, Figure S5 (right). The SAXS data for the four contrasts are identical, implying no isotopic effects and that the underlying structure is the same. However, the SANS patterns are all different; scattering curves from HD-DES with d-dodecane and HH-DES with h-dodecane are dominated by the characteristic broad peak at *q* ∼ 0.2 Å^–1^, whereas HD-DES with h-dodecane shows micellar scattering superposed with a peak like feature at *q* ≈ 0.25 Å^–1^ and the HH-DES with d-dodecane shows micellar scattering with a structure factor with a bump like feature at *q* ≈ 0.3 Å^–1^. The SANS data from HD-DES with d-dodecane and HH-DES with h-dodecane were fitted to the broad peak model and give the same *d*-spacing within an experimental resolution of 30.7 Å. The SAXS data from all samples were also fitted to the broad peak model and give the same *d*-spacing within experimental resolution of 29.4 Å. The 1 Å difference in the calculated *d*-spacing between SANS and SAXS could arise from the differences in visible contrast between the two techniques, with SANS seeing the alkyl nonpolar domains better and is not considered significant. It was not possible to fit the four SANS contrasts to a single model, so we simulated a core–shell prolate ellipsoid with a hard sphere structure factor to model the data. The core in this case comprises 1.5 × sulfonic acid with sulfobetaine headgroup, the shell comprises 1.5 × camphor with 1/3rd (C4) sulfobetaine alkyl chain, and the solvent comprises the 2/3rd (C8) sulfobetaine alkyl chain with the dodecane. Using reasonable estimates for core radius (16 and 7 Å) and shell thickness (5 Å), we are able to within reason reproduce the details of 3 contrasts (HD-DES with d-dodecane, HH-DES with h-dodecane, and HH-DES with d-dodecane); however, we still cannot capture the details of the fourth contrast (HD-DES with h-dodecane). Fitting the HD-DES with h-dodecane to a simple ellipsoidal model with a broad peak gives the ellipsoid radii as 22 and 8 Å and a peak with *d*-spacing of 23.5 Å. The ellipsoid dimensions are consistent with the simulation we created for the other three data sets. We also do not understand the origin of the broad peak at *q* ≈ 0.25 Å^–1^. The details of the model and fitting are provided in Supporting Information Section S3.7 and Figure S9.

**Figure 7:**
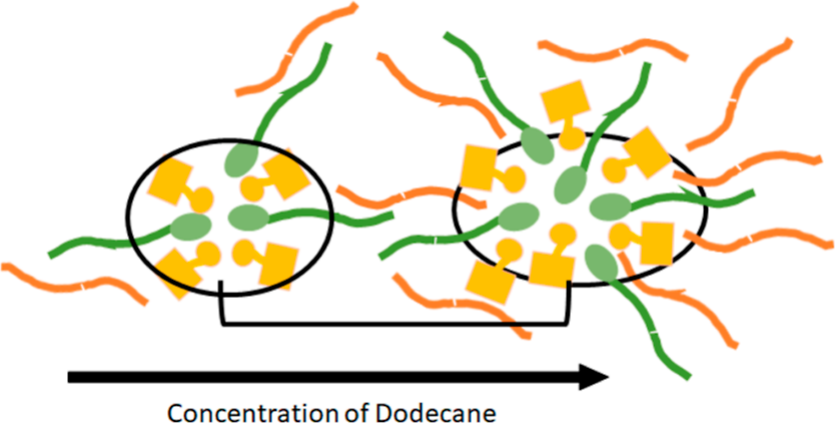
A schematic depicting the proposed DES structure with the CSA shown as yellow blocks, the sulfobetaine shown in green, and the dodecane in orange. The black bracket depicts the repeating unit that gives rise to the *d*-spacing.

The data and simulations suggest that dodecane does not just incorporate in the DES structure but instead interacts with the alkyl chains and leads to an inverse ellipsoidal micellar arrangement of the polar (sulfonic acid and sulfobetaine headgroup) and nonpolar domains (camphor, sulfobetaine tails and now dodecane) in the DES structure. The interaction of the dodecane with the alkyl chain effectively increases the nonpolar domain volume, such that now the packing parameter is >1 and structures with a curvature are favored leading to formation of inverse micelles, which as the dodecane concentration increases (nonpolar domain volume increases), become more elongated. A schematic for this is shown in Figure 7. This local inverse micellar arrangement means that the overall structure of the DES + dodecane mixture changes from a bicontinuous sponge-like to close packing of the inverse ellipsoidal micelles. The broad peak in certain SANS contrasts could be related to a smeared out structure factor due to the dispersity of distances in the structure. This quasi-micellar organization also corresponds with the observed shear thinning behavior, since concentrated inverse micellar phases in surfactant–water systems have previously been demonstrated to align to a more anisotropic/elongated arrangement when sheared, which leads to a non-Newtonian shear thinning behavior.^50,51^

## Conclusions

We have investigated nanostructure and flow properties in the amphiphile-based CSA:SB3-Cn DES. The DES shows nanostructuring on 2–3 nm length scale with local lamellar arrangement of alternating polar (sulfobetaine headgroup and CSA) and apolar (sulfobetaine alkyl chains) domains, where the domain size increases linearly with the alkyl chain length. The overall structure we propose is a disordered bicontinuous sponge-like phase, similar to that observed for SAILS.^39−41^ The nonpolar regions of the sponge-like phase are composed of the short camphor units of the CSA, between the longer, interdigitated sulfobetaine alkyl chains, which could act to restrict flow within the nonpolar phase. The sulfobetaine and CSA headgroups in the polar region are also constrained by electrostatic interactions between these moieties in the absence of water. This nanostructuring results in a highly viscous DES with a viscosity of ∼5 Pa s at 70 °C.

Interaction of the DES with polar and nonpolar moieties affects both the structure and flow properties of the DES. Water incorporates into the polar domains in the DES structure and without disrupting the bicontinuous sponge-like structure leads to a swelling of the lamellar domains (increased *d*-spacing). A similar swelling is also observed for [BMIm][AOT] SAIL upon the addition of small amounts of water (up to 20 vol %).^49^ This swelling of the structure by the water fluidizes the polar regions, solvating these species, and reducing direct interactions between the polar sulfobetaine and CSA moieties. This leads to a drastic reduction in viscosity with concentration of water added such that 20 wt % added water reduces the viscosity of the DES by an order of magnitude. Dodecane on the other hand not only swells the DES structure by incorporating into the alkyl chains but also leads to an inverse ellipsoidal micellar arrangement of the polar (sulfonic acid and sulfobetaine headgroup) and nonpolar domains (camphor, sulfobetaine tails, and dodecane) in the DES structure, which at high dodecane concentration results in random close packing of the inverse ellipsoidal micelles. In the case of dodecane, the viscosity change is concentration dependent. At low dodecane concentrations, the viscosity is reduced though not to the same extent as that with water, due to dodecane chains, which are not pinned to the polar–nonpolar interface, fluidizing the nonpolar region. However, at high dodecane concentration a strong non-Newtonian behavior is observed for the DES + dodecane mixture, with the low shear viscosity being an order of magnitude more than that of the neat DES, and which finally decreases to viscosity lower than that of the neat DES. We suggest that this arises due to the formation of close-packed (although disordered) inverse ellipsoidal micelles upon addition of high concentrations of dodecane, since similar rheological behavior is often also observed for more ordered cubic inverse micellar liquid crystalline phases.^50,51^ It would be interesting to ascertain if the long chain structure of the solute is responsible for the non-Newtonian behavior and the structural disruption caused by dodecane. To investigate this, a comparative study with varying chain length alkanes could be undertaken.

The CSA:SB3-Cn DES shows a nanostructure with polar and apolar domains. We have demonstrated the potential of this nanoorganization to interact with polar or nonpolar moieties. This can play an important role for new applications such as smart synthesis, solute confinement, tunable phase equilibria, as well as understanding additives as flow modifiers to lower DES viscosity in practical applications. The observed nanostructure organization will require further detailed theoretical and experimental efforts to be fully understood and exploited.

## Supporting Information Available

DSC and viscosity measurements for the CSA:SB3-Cn DES; SAXS, WAXS, and SANS data for the DES and DES with added water and dodecane; and details of the model used to fit the data and scattering length density calculations for the various components (PDF)
