# Supplementary material for: Nanostructure in Amphiphile-Based Deep Eutectic Solvents
Source: Langmuir. 2023 Nov 15;39(47):16776–84. doi: 10.1021/acs.langmuir.3c02105 (PMC10688184; doi:10.1021/acs.langmuir.3c02105)
Supplement: Supplementary file 1 — la3c02105_si_001.pdf [file la3c02105_si_001.pdf]

Supporting Information

Nanostructure in Amphiphile-Based Deep Eutectic Solvents

Iva Manasi,*† Ralf Schweins‡, Kun Ma¶ and Karen J. Edler*§†

†*Department of Chemistry, University of Bath, Claverton Down, Bath BA2 7AY, UK; E-mail: im554@bath.ac.uk*

‡*Institut Laue-Langevin, CS 20156, 38042 Grenoble Cedex 9, France*

¶*ISIS Neutron and Muon Source, STFC, Rutherford Appleton Laboratory, Didcot, OX11 0QX, UK*

§*Department of Chemistry, Centre for Analysis and Synthesis (CAS) Lund University, Lund, 221 00 Sweden; E-mail: karen.edler@chem.lu.se; Phone: +46(0)46-2228734*

S1 Differential scanning calorimetry (DSC) measurements

DSC measurement carried out on the CSA:SB3-Cn DES are shown in Figure S1. The difficulty of measuring a DES glass transition temperature (T_g) using DSC has been covered in the literature; melting points are challenging to reproduce in DES due to supercooling, and cooling rate and sample environment (i.e. confinement) effects, [1, 2, 3] as well as instrumental limitations. The DSC measurements are shown in Figure S1 and are relatively free of thermal events. However, there is a subtle baseline shift with onset of ~ -20 °C on both cooling and heating for all 4 DES samples (CSA:SB3-12, CSA:SB3-14, CSA:SB3-16 and CSA:SB3-18). This is consistent with the slight change in specific heat capacity upon glass transition. Using these measurements a T_g of -19.4 °C can be estimated for CSA:SB3-12, -20.8 °C for CSA:SB3-14, -22.5 °C for CSA:SB3-16 and -18.5 °C for CSA:SB3-18.

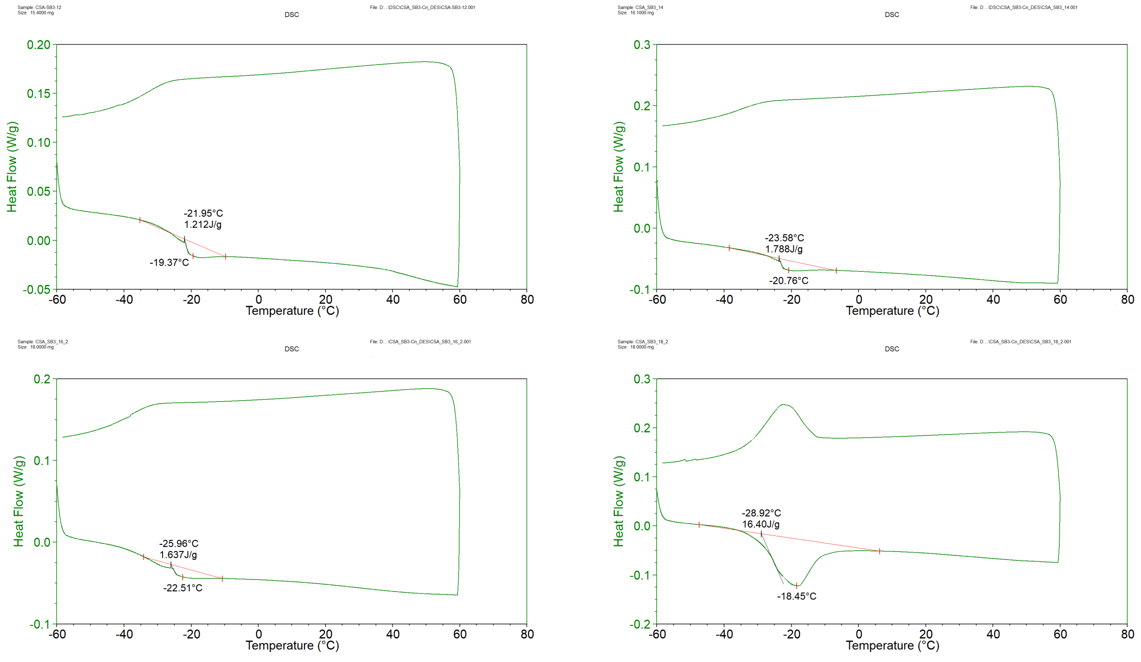

Figure S1: Differential scanning calorimetry (DSC) measurements for CSA:SB3-Cn DES: CSA:SB3-12 (top left), CSA:SB3-14 (top right), CSA:SB3-16 (bottom left), and CSA:SB3-18 (bottom right). The samples were first equilibrated at -60 °C and held for 5 min, heated to 60 °C at a ramp rate of 5 °C min^{-1} and held for 5 mins, before cooling to -60 °C at the same ramp rate.

S2 Viscosity measurements

Viscosity measurements were carried out for the CSA:SB3-Cn DES comprising different alkyl chain lengths sulfobetaine for shear rates ranging from 0.01 – 100 s^{-1} at a temperature of 70 °C. As can be seen from the graph there is no shear rate dependence of the viscosity for either of the 4 DES. The average viscosity for the DES are 4.6 ± 0.11 Pa s for CSA:SB3-12, 4.5 ± 0.10 Pa s for CSA:SB3-14, 5.5 ± 0.12 Pa s for CSA:SB3-16 and 6.0 ± 0.19 Pa s for CSA:SB3-18.

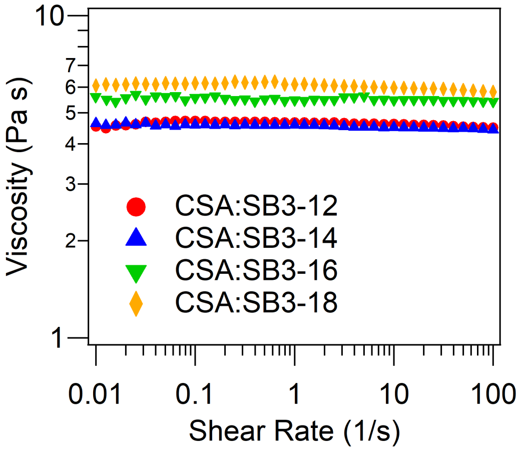

Figure S2: Viscosity vs shear rate for the CSA:SB3-Cn DES for different alkyl chain lengths sulfobetaine: SB3-12 (red data set), SB3-14 (blue data set), SB3-16 (green data set) and SB3-18 (yellow data set).

Viscosity measurements were also carried out for the CSA:SB3-12 and CSA:SB3-12 DES with added water and added dodecane for shear rates ranging from 0.01 – 100 s^{-1} at a temperature of 70 °C. Three different concentrations of water/dodecane were measured 5 wt%, 10 wt% and 20 wt%. The data is shown in top left panel in Figure 4 and Figure 5. Table S1 summarises the viscosity values for the DES with added water/dodecane at 3 different values of shear rate: low shear (0.01 s^{-1}), high shear rate (10 s^{-1}) and very high shear rate (100 s^{-1}) and Figure S3 shows the high shear (10 s^{-1}) viscosity vs concentration of the water/dodecane for the two DES.

Table S1: Viscosity values for the CSA:SB3-12 & CSA:SB3-18 DES with added water and dodecane at 3 different values of shear rate: low shear (0.01 s^{-1}), high shear rate (10 s^{-1}) and very high shear rate (100 s^{-1}).

Sample	Viscosity at different shear rate (Pa s)					
	0.01 s^{-1}	10 s^{-1}	100 s^{-1}	0.01 s^{-1}	10 s^{-1}	100 s^{-1}
	CSA:SB3-12 DES			CSA:SB3-18 DES		
Neat DES	4.97	4.68	4.54	6.06	5.97	5.8
+ 5% Water	2.07	1.54	1.51	2.35	1.77	1.74
+ 10% Water	0.79	0.79	0.78	1.46	1.16	1.12
+ 20% Water	0.62	0.46	0.44	0.91	0.74	0.72
+ 5% Dodecane	4.05	3.08	2.94	4.47	3.55	3.43
+ 10% Dodecane	2.56	2.29	2.23	4.8	3.6	3.49
+ 20% Dodecane	0.57	0.45	0.43	41.41	3.7	2.67

HD DES is h-CSA:d-SB3-12 and HH DES is h-CSA:h-SB3-12.
d-DD d-dodecane and h-DD is h-dodecane.

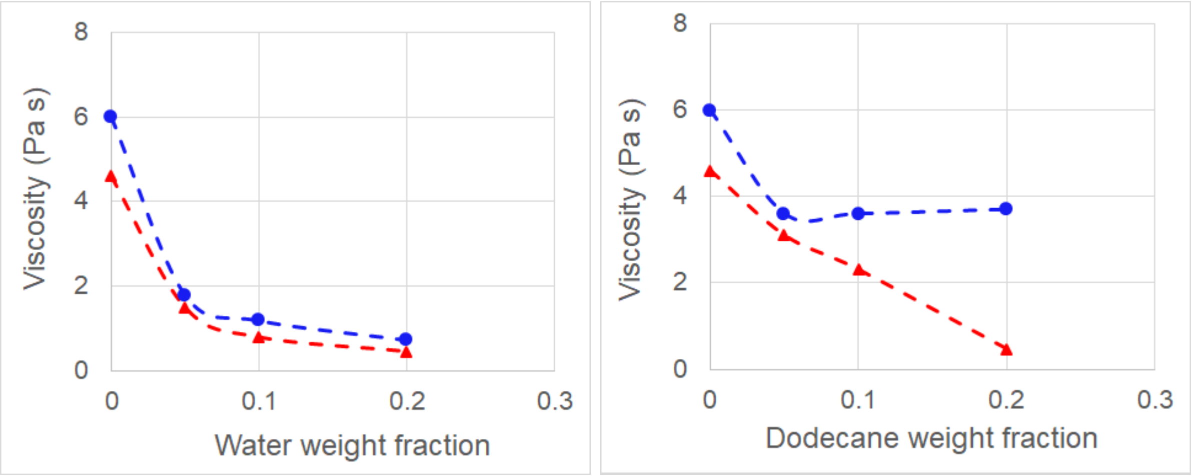

Figure S3: High shear (10 s^{-1}) viscosity vs concentration of water (left) and dodecane (right) for CSA:SB3-12 (red triangles) and CSA:SB3-18 (blue circles). The trendline shows a guide to the eye.

S3 SAXS and SANS measurements

S3.1 SLD values of various components in the system

The scattering length density (SLD) of any component in a system is calculated using the equation:

$$\rho = \frac{\sum_i^n b_{c_i}}{\overline{V}} \tag{S1}$$

where b_{c_i} is the bound coherent scattering length of atom i in a molecule and $\overline{V}$ is the volume containing all the n atoms. The molar volumes, neutron scattering lengths and calculated SLD for various individual components in the system are given in Table S2.

Table S2: Volumes, neutron scattering lengths ($\sum b_{c_i}$) and calculated SLD of constituents of the system. The neutron scattering length of each constituent was calculated as the sum of the neutron scattering lengths of the individual atoms in the unit.

Chemical	Chemical Formula	Volume	$\sum b_{c_i}$	SLD
		Å^3	fm	$\times 10^{-06}$ Å^{-2}
Camphor Sulfonic Acid	$C_{10}H_{16}O_4S$	303.7	32.8	1.08
Sulfonic Acid	SO_3H	59.26	16.53	2.79
Camphor	$C_{10}H_{15}O$	253.7	16.24	0.64
SB3-Cn head	$C_5H_{12}NO_3S$	255	16.32	0.9
C12 alkyl tail	$C_{12}H_{25}$	377.13	-13.58	-0.36
d25-C12 alkyl tail	$C_{12}D_{25}$	377.13	246.64	6.54
C14 alkyl tail	$C_{14}H_{29}$	430.13	-15.48	-0.36
d29-C14 alkyl tail	$C_{14}D_{29}$	430.13	286.47	6.66
C16 alkyl tail	$C_{16}H_{33}$	486.16	-17.02	-0.35
C18 alkyl tail	$C_{18}H_{37}$	541.75	-18.96	-0.35
Water	H_2O	30	-1.68	-0.56
d-Water	D_2O	30	19.14	6.38
Dodecane	$C_{12}H_{26}$	377.13	-17.35	-0.46
d26-Dodecane	$C_{12}H_{26}$	377.13	253.43	6.72

The molar volume of various components was calculated using their molecular composition and density from online databases.

S3.2 Models used for fitting SAXS and SANS data

The SAXS and SANS data in this work were fitted to the broad peak form factor. This is an empirical forms factor for SANS/SAXS data characterized by a broad scattering peak, such as that observed from our samples. The scattering intensity is given by: [4]

$$I(q) = \frac{A}{Q^n} + \frac{C}{A + (|q - q_0|\xi)^m} + B \tag{S2}$$

where the first term accounts for the Porod scattering in the system (low-q power law in our case), B is the incoherent background and the second term accounts for the characteristic scattering from the inhomogeneities in the system giving rise to the broad peak. Here C is the Lorentz scale factor, m is the Lorentz exponent (fixed at 2 for our data), ξ is the Lorentz screening length calculated from the width of the peak, and q_0 is the peak position and is related to the d-spacing as $q_0 = 2\pi/d$. The d-spacing is the characteristic distance between the scattering inhomogeneities such as in lamellar, cylindrical, or spherical morphologies or for bicontinuous structures.

In our system the low q ($q < 0.01$ Å^{-1}) can be ascribed to the presence of residual micro air bubbles in this highly viscous DES. The scattering shows a $\sim q^{-4}$ dependence in all the systems, no trend with alkyl chain length or additive concentration, and is is lower in systems with more hydrogens, which supports this hypothesis. The broad peak in our system arises due to the domains formed as a result of the CSA and SB3-Cn structural arrangement in the DES.

S3.3 SAXS data from CSA:SB3-Cn DES

This section details the complementary SAXS data from CSA:SB3-Cn DES (SB3-12, SB3-14, SB3-16 and SB3-18) and for SB3-12 same composition but different SANS contrast samples. The SAXS data is collected on the same samples as the SANS data. Figure S4 left panel is complementary to left panel in Figure 2, Figure S4 right panel is complementary to right panel in Figure 2 and Figure S5 is complementary top left panels in Figure 4 and 5. The data from same composition but different contrast SANS samples (Figure S4 right panel and Figure S5) are identical within measurement resolution and confirm that there is no isotope effect on the structure in the samples.

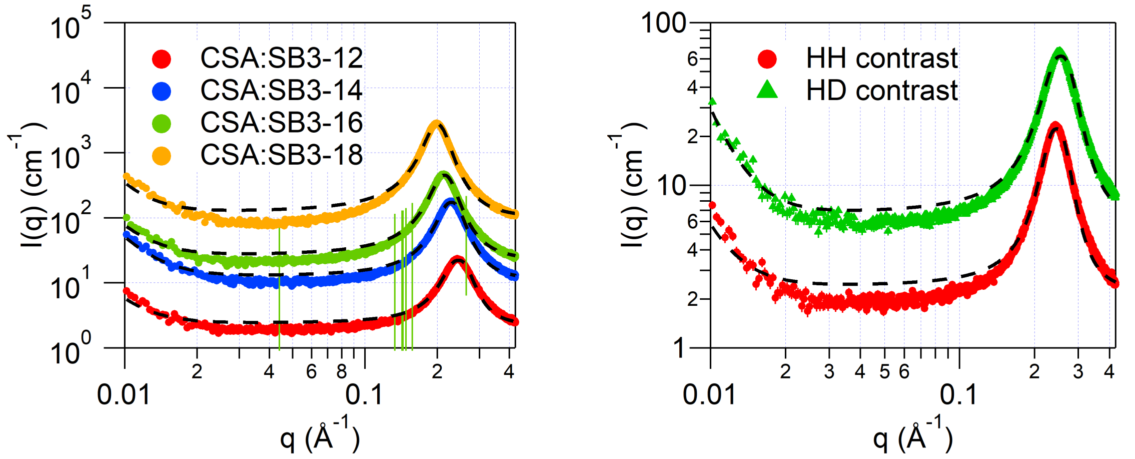

Figure S4: Left panel: SAXS data from CSA-SB3-Cn DES comprising different alkyl chain lengths sulfobetaine with the CSA:SB3-12 shown in red, CSA:SB3-14 in blue, CSA:SB3-16 in green and CSA:SB3-18 in yellow. Right panel: SAXS data from CSA-SB3-12 DES with HH contrast (red data set) and HD contrast (green data set). The data is fitted to a broad peak model (black dashed lines). The various SAXS traces in the figures are offset along the y-axis for clarity.

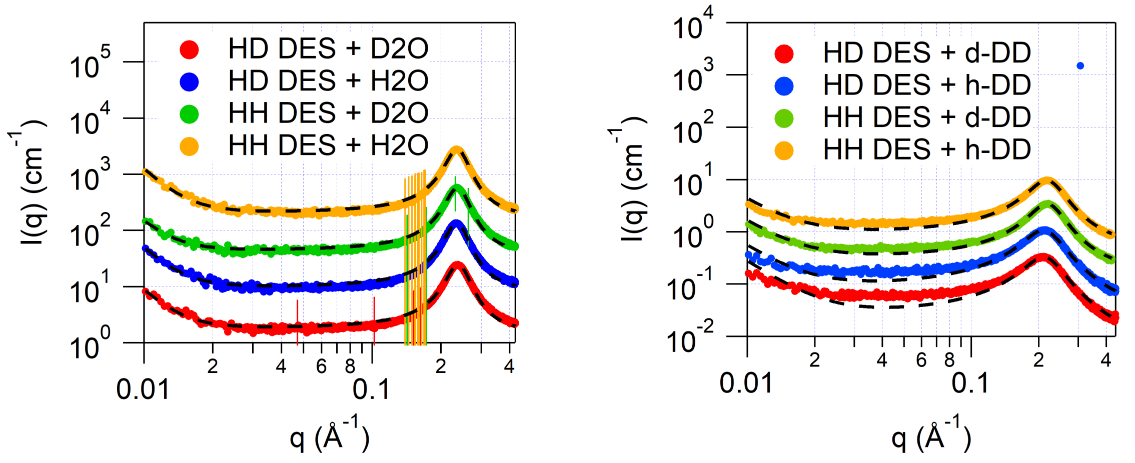

Figure S5: SAXS data from 10 wt% added water (Left panel) and 10 wt% added dodecane in CSA:SB3-12 at 4 different contrasts: HD DES with D_2O/d-dodecane (red), HD DES with H_2O/h-dodecane (blue), HH DES with D_2O/d-dodecane (green) and HH DES with H_2O/h-dodecane (yellow). The data is fitted to a broad peak model (black dashed lines). The various SAXS traces in the figures are offset along the y-axis for clarity.

S3.4 WAXS data from CSA:SB3-Cn DES

This section details the WAXS data from CSA:SB3-Cn DES without and with 10 wt% added water/dodecane collected at the same time as the SAXS and on the same samples as the SANS data. Figure S6 left panel shows the WAXS data from the neat CSA:SB3-cn DES for the different alkyl chain lengths of the SB3-Cn (SB3-12, SB3-14, SB3-16 and SB3-18) and the right panel shows the WAXS data from the CSA:SB3-12 DES with 10 wt% added water and dodecane. The curves show no higher order peaks related to the first peak. A broad scattering feature exists between 1.2 – 1.5 Å^{-1}, which is a typical "adjacency" peak arising from overall intermolecular and intramolecular interactions between adjacent atoms that exists in all fluids regardless of their polarity. [5, 6]

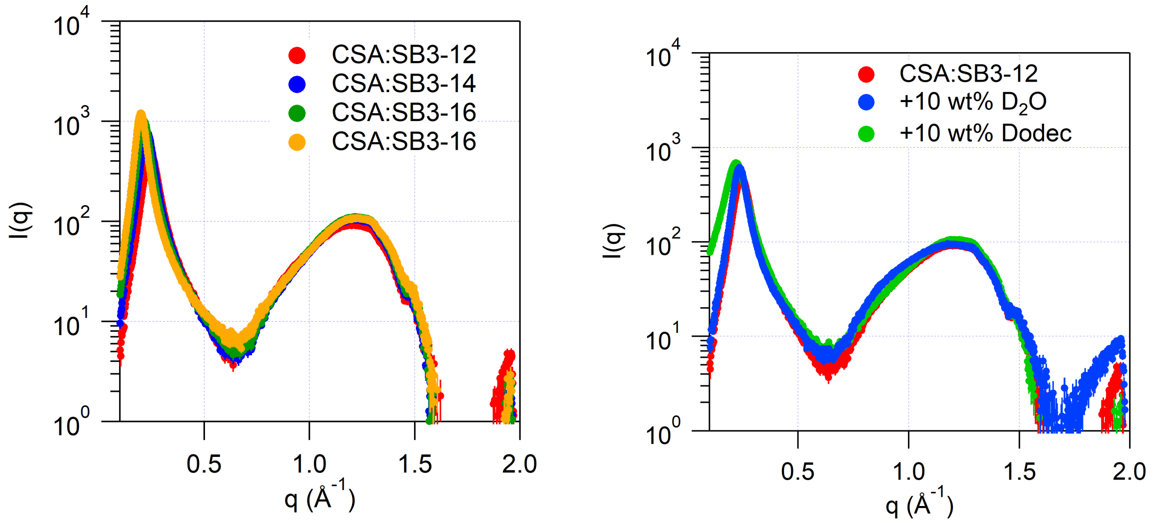

Figure S6: Left panel: WAXS data from CSA-SB3-Cn DES comprising different alkyl chain lengths sulfobetaine with the CSA:SB3-12 shown in red, CSA:SB3-14 in blue, CSA:SB3-16 in green and CSA:SB3-18 in yellow. Right panel: WAXS data from CSA-SB3-12 DES (red data set), DES with 10 wt% added D_2O (blue data set) and DES with 10 wt% added d-dodecane (green data set).

S3.5 SANS data from CSA:SB3-Cn DES with water

This section details the SANS data from h-CSA:h-SB3-Cn (SB3-14, SB3-16 and SB3-18) with D2O at 3 different concentrations: 5 wt%, 10 wt% and 20 wt%. This is complementary to the SANS data from h-CSA:h-SB3-12 with D2O shown in Figure 4 (bottom left).

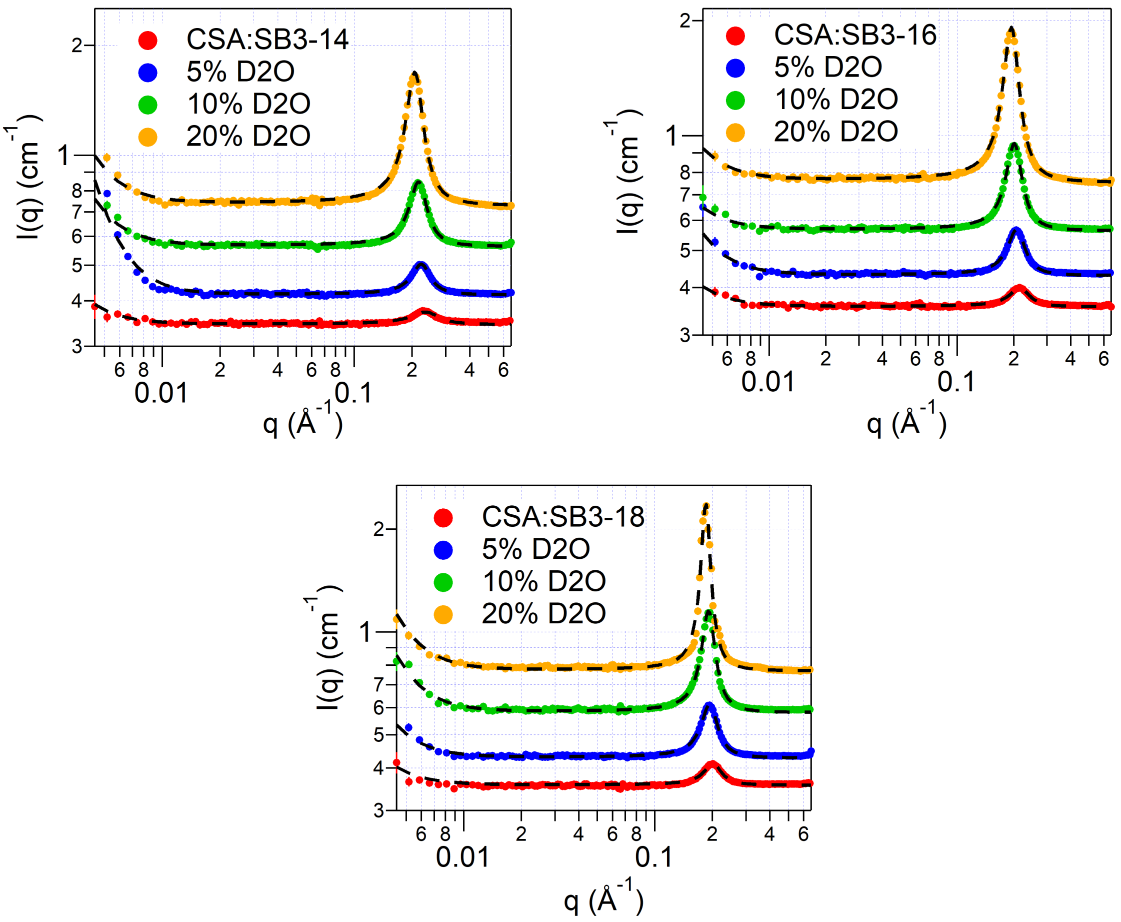

Figure S7: SANS data from CSA:SB3-14 (top left), CSA:SB3-16 (rop right) and CSA:SB3-18 (bottom) DES with added D_2O at 3 concentrations: 5 wt% (blue data set), 10 wt% (green data set) and 20 wt% (yellow data set) along with the neat DES (red data set). The various SANS traces in the figures are offset along the y-axis for clarity.

S3.6 SAXS data from CSA:SB3-Cn DES with dodecane

This section details the SANS data from h-CSA:h-SB3-Cn (SB3-14, SB3-16 and SB3-18) with d-dodecane at 3 different concentrations: 5 wt%, 10 wt% and 20 wt%. This is complementary to the SANS data from h-CSA:h-SB3-12 with d-dodecane shown in Figure 6 (bottom left).

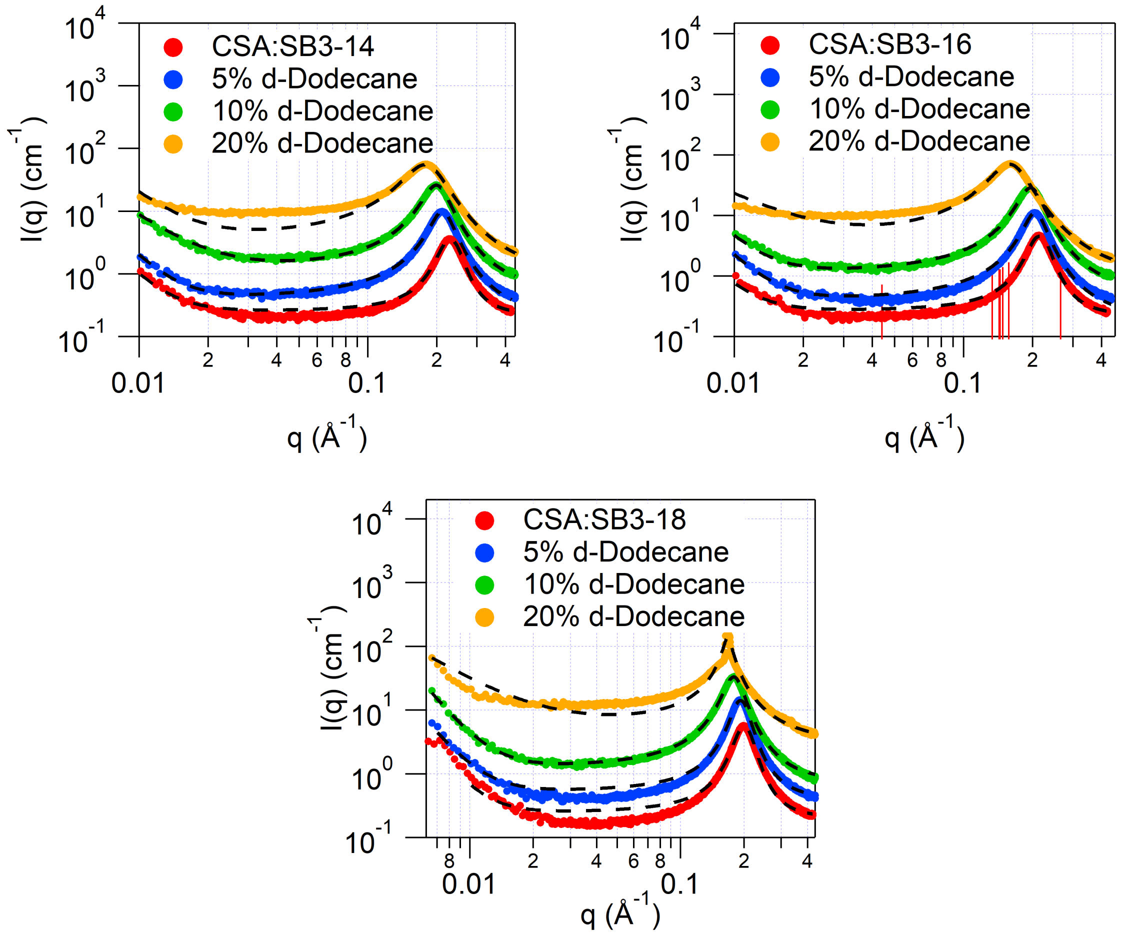
5% d-Dodecane
10% d-Dodecane
20% d-Dodecane
I(q) (cm^{-1})
q (Å^{-1})
5% d-Dodecane
10% d-Dodecane
20% d-Dodecane
I(q) (cm^{-1})
q (Å^{-1})

Figure S8: SAXS data from CSA:SB3-14 (top left), CSA:SB3-16 (rop right) and CSA:SB3-18 (bottom) DES with added d-dodecane 3 concentrations: 5 wt% (blue data set), 10 wt% (green data set) and 20 wt% (yellow data set) along with the neat DES (red data set). The various SAXS traces in the figures are offset along the y-axis for clarity.

S3.7 Fit model and parameters to simulate multi-contrast SANS data from CSA:SB3-12 + 10 wt% dodecane

Simulated data for the SANS from CSA:SB3-12 with 10 wt% dodecane (shown in Figure 6 top right) at different contrasts (HD DES + d-dodecane, HD DES + h-dodecane, HH DES + d-dodecane and HH DES + h-dodecane) is shown in Figure S9. The simulation model comprises core-shell prolate ellipsoid with a hard sphere structure factor. The scattered intensity, $I(q)$, of a system of monodisperse, isotropic, centrosymmetric particles may be described by the equation:

$$I(q) = scale\frac{V_f}{V}P(q)S(q) + B \tag{S3}$$

where V_f and V is volume fraction and volume of the particles, respectively, $P(q)$ refers to the form factor, which describes scattering within the particle and therefore relates to the particle shape, and $S(q)$ is the structure factor, which describes the interaction between scattering particles in the system. A detailed description along with the mathematical models of the structure factor and form factor for ellipsoidal along with its core-shell variant scattering geometries can be found in [7, 4, 8, 9, 10].

In the simulation, we used an ellipsoid radii of 16 Å and 7 Å, a shell thickness of 5 Å and a volume fraction of the scatters of 0.2. The core consists of the sulfonic acid and SB3-12 headgroup in the molar ratio 1.5:1, the shell comprises camphor (from the CSA), 1/3rd the SB3-12 alkyl chain in the molar ratio 1.5:1 and the solvent comprises the remaining 2/3rd of the SB3-12 alkyl chain and the dodecane in the molar ratio 2.5:1 (calculated using the solvent mixture composition). The SLDs for this core-shell structure were calculated using component SLDs from Table S2 and are given in Table S3.

This model represents the SANS data from HD DES + d-dodecane, HH DES + d-dodecane and HH DES + h-dodecane well. For the HD DES + h-dodecane we use a simple ellipsoidal model with a broad peak.

Table S3: SLD calculation for the core-shell prolate ellipsoid model used to simulate the SANS data for CSA:SB3-12 with dodecane at different contrasts as shown in Figure S9.

Contrast	Core 1.5*Sulfonic Acid + 1*SB3 head $\times 10^{-06}$ Å^{-2}	Shell 1.5*camphor + 1*1/3rd SB3 tail (C4) $\times 10^{-06}$ Å^{-2}	Solvent 2.5*Sb3 tail (C8) + 1*Dodecane $\times 10^{-06}$ Å^{-2}
HD DES + d-DD	1.52	2.06	6.66
HD DES + h-DD	1.52	2.06	3.97
HH DES + d-DD	1.52	0.42	2.26
HH DES + h-DD	1.52	0.42	-0.43

HD DES is h-CSA:d-SB3-12 and HH DES is h-CSA:h-SB3-12.
d-DD d-dodecane and h-DD is h-dodecane.

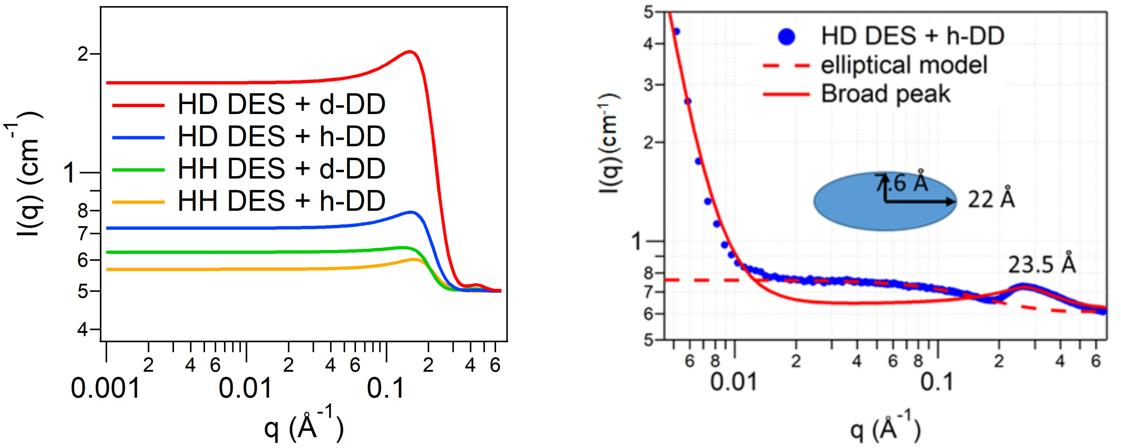

Figure S9: Left panel: Simulated SANS data for core-shell prolate ellipsoid with a hard sphere structure factor to model the CSA:SB3-12 with dodecane at different contrasts (HD DES + d-dodecane; red, HD DES + h-dodecane; blue, HH DES + d-dodecane; green, HH DES + h-dodecane; yellow). The SLDs used in the model are given in Table S3. Right panel: SANS data from HD DES + h-dodecane fitted to a prolate ellipsoid and a broad peak model.

References

[1] María Francisco, Adriaan van den Bruinhorst, Lawien F. Zubeir, Cor J. Peters, and Maaike C. Kroon. A new low transition temperature mixture (LTTM) formed by choline chloride+lactic acid: Characterization as solvent for CO2 capture. *Fluid Phase Equilibria*, 340:77 – 84, 2013.

[2] Oliver S. Hammond, Daniel T. Bowron, Andrew J. Jackson, Thomas Arnold, Adrian Sanchez-Fernandez, Nikolaos Tsapatsaris, Victoria Garcia Sakai, and Karen J. Edler. Resilience of malic acid natural deep eutectic solvent nanostructure to solidification and hydration. *J. Phys. Chem. B*, 121(31):7473–7483, 2017. PMID: 28699758.

[3] Iva Manasi, Mohammad R. Andalibi, Ria S. Atri, Jake Hooton, Stephen M. King, and Karen J. Edler. Self-assembly of ionic and non-ionic surfactants in type iv cerium nitrate and urea based deep eutectic solvent. *J. Chem. Phys.*, 155(8):084902, 2021.

[4] SasView for Small Angle Scattering Analysis, Version: 5.0.4, 2021.

[5] Hemant K. Kashyap, Cherry S. Santos, Harsha V. R. Annapureddy, N. Sanjeeva Murthy, Claudio J. Margulis, and Edward W. Castner, Jr. Temperature-dependent structure of ionic liquids: X-ray scattering and simulations. *Faraday Discuss.*, 154:133–143, 2012.

[6] Juan C. Araque, Jeevapani J. Hettige, and Claudio J. Margulis. Modern room temperature ionic liquids, a simple guide to understanding their structure and how it may relate to dynamics. *J. Phys. Chem. B*, 119(40):12727–12740, 2015. PMID: 26244375.

[7] Steven R. Kline. Reduction and analysis of SANS and USANS data using IGOR Pro. *J. Appl. Crystallogr.*, 39(6):895–900, Dec 2006.

[8] Michael Kotlarchyk and Sow-Hsin Chen. Analysis of small angle neutron scattering spectra from polydisperse interacting colloids. *J. Chem. Phys.*, 79(5):2461–2469, 09 1983.

[9] Jan Skov Pedersen. Analysis of small-angle scattering data from colloids and polymer solutions: modeling and least-squares fitting. *Advances in Colloid and Interface Science*, 70:171–210, 1997.

[10] Ingo Breßler, Joachim Kohlbrecher, and Andreas F. Thünemann. *SASfit*: a tool for small-angle scattering data analysis using a library of analytical expressions. *Journal of Applied Crystallography*, 48(5):1587–1598, Oct 2015.
